# DHPLC technology for high-throughput detection of mutations in a durum wheat TILLING population

**DOI:** 10.1186/s12863-016-0350-0

**Published:** 2016-02-17

**Authors:** Pasqualina Colasuonno, Ornella Incerti, Maria Luisa Lozito, Rosanna Simeone, Agata Gadaleta, Antonio Blanco

**Affiliations:** Department of Soil, Plant and Food Sciences, section of Genetic and Plant Breeding, University of Bari “Aldo Moro”, Via G. Amendola 165/A, 70126 Bari, Italy

**Keywords:** Durum wheat, TILLING, SNP markers, DHPLC

## Abstract

**Background:**

Durum wheat (*Triticum turgidum* L.) is a cereal crop widely grown in the Mediterranean regions; the amber grain is mainly used for the production of pasta, couscous and typical breads. Single nucleotide polymorphism (SNP) detection technologies and high-throughput mutation induction represent a new challenge in wheat breeding to identify allelic variation in large populations. The TILLING strategy makes use of traditional chemical mutagenesis followed by screening for single base mismatches to identify novel mutant loci. Although TILLING has been combined to several sensitive pre-screening methods for SNP analysis, most rely on expensive equipment. Recently, a new low cost and time saving DHPLC protocol has been used in molecular human diagnostic to detect unknown mutations.

**Results:**

In this work, we developed a new durum wheat TILLING population (cv. Marco Aurelio) using 0.70-0.85 % ethyl methane sulfonate (EMS). To investigate the efficiency of the mutagenic treatments, a pilot screening was carried out on 1,140 mutant lines focusing on two target genes (Lycopene epsilon-cyclase, *ε-LCY*, and Lycopene beta-cyclase, *β-LCY*) involved in carotenoid metabolism in wheat grains. We simplify the heteroduplex detection by two low cost methods: the enzymatic cleavage (*Cel*I)/agarose gel technique and the denaturing high-performance liquid chromatography (DHPLC). The *Cel*I/agarose gel approach allowed us to identify 31 mutations, whereas the DHPLC procedure detected a total of 46 mutations for both genes. All detected mutations were confirmed by direct sequencing. The estimated overall mutation frequency for the pilot assay by the DHPLC methodology resulted to be of 1/77 kb, representing a high probability to detect interesting mutations in the target genes.

**Conclusion:**

We demonstrated the applicability and efficiency of a new strategy for the detection of induced variability. We produced and characterized a new durum wheat TILLING population useful for a better understanding of key gene functions. The availability of this tool together with TILLING technique will expand the polymorphisms in candidate genes of agronomically important traits in wheat.

**Electronic supplementary material:**

The online version of this article (doi:10.1186/s12863-016-0350-0) contains supplementary material, which is available to authorized users.

## Background

Traditional chemical induced mutations together with the modern SNP detection technologies are an attractive mixture of procedures to explore gene function and for the production of phenotypic variants in traits of agronomic interest, especially in species with complex genomes such as grasses [1].

The Targeting Induced Local Lesions IN Genomes (TILLING) technique is a mutational approach widely exploited to screen for single base changes induced by chemical mutagenesis (usually ethyl methane sulfonate, EMS) in target genes [2–4]. The main effort is to analyze large mutant populations through DNA samples organized in pools, followed by mutant pool detection and validation of the individual carrying the mutation by sequencing. The recent technological advances have led to improve the performance in cost and time allowing to combine traditional mutation programs to high-throughput pre-screening techniques [5–9].

TILLING can be carried out using different procedures, such as the heteroduplex mismatch cleavage assay through single-strand-specific endonuclease *Cel*I [10], the DNA melting temperature analysis [11] and the deep sequencing of pooled DNA [12]. The *Cel*I assay represents the most popular, accessible and low-cost method to detect mutations in large populations [13]. It has been combined with agarose gel detection approaches [14–16], with a non-denaturing polyacrylamide method [9, 17] or a gel based system (LI-COR DNA analyzer) requiring labeled PCR primers [10, 18].

The mutation scanning High Resolution DNA Melting (HRM) method takes advantage of the denaturing dynamics of double-stranded DNA after PCR amplification and heteroduplex formation. The single base changed in mutant lines is detected by observing a slight shift in the melting temperature compared to the wild-type homoduplexes. Other pre-screening methods for SNP detection based on DNA melting temperature, such as Denaturing Gradient Gel Electrophoresis (DGGE) [19], Single-Strand Conformational Polymorphism (SSCP) [20] and Mass Spectrometry (MS) [21], were found to have limitations in terms of sensitivity, costs and suitability for high-throughput screening.

Although the sequencing is the direct method to reveal mutations, its application is beyond the budget of low-to-medium-throughput academic laboratories. In the last few years, the progresses of Next-Generation Sequencing (NGS) technologies have helped to bypass some limits of the conventional TILLING approaches such as SNP detection in pools with more than eight individuals [22]. The NGS progresses have converted the TILLING strategy into an *in silico* procedure denominated TILLING by Sequencing (TbyS) [12]. This approach has been performed considering the complete coding region of each mutant genome (exome) organized in a multidimensional pool system [23]. Despite the several advantages of TbyS combined with Exome Capture strategy, its applicability continues to be restricted for the money input required per single capture reaction and the genome complexity of crop species.

A recently developed and powerful technique, commonly referred to as Denaturing High Performance Liquid Chromatography (DHPLC), allows the detection of single base substitutions as well as small insertions and/or deletions in a large number of samples [24]. In brief, this method detects mismatches through heteroduplex formation between wild-type and mutated DNA strands. Heteroduplex molecules are obtained by mixing, denaturing and re-annealing the PCR products; the samples are then separated by reverse-phase liquid chromatography on a special column matrix with partial heat denaturation of DNA strands [25]. So far, DHPLC is mainly used in human diagnosis to detect mutations involved in several diseases [26, 27] and to assess antibiotic resistance mutations in microbial communities [28]. In plants, DHPLC has been applied in the analysis of natural variations [29], varietal traceability [30] and candidate gene studies [31]. As a pre-screening method in TILLING strategy, DHPLC has been first adopted in *Arabidopsis thaliana* [32] through melting temperature analysis and in barley [33] in combination with *Cel*I endonuclease.

Durum wheat [*Triticum turgidum* (L.) subsp. *durum* (Desf.) Husn.] represents one of the most challenging candidates for the TILLING technology because of its genome characteristics, such as the presence of genes in two homoeologous copies (in A and B genomes) sharing ~93-96 % sequence identity, the large genome size (~13,000 Mb), the high portion of repetitive DNA (83 %) and the low gene density [34]. In this vein, the TILLING strategy is important for reverse genetic studies and for the genetic improvement of wheat. It is applied in wheat as functional and/or alternatively to non-genetically manipulation (GM) technique.

Here we report the development of a durum wheat TILLING population and its characterization through TILLING strategy analyzing two target genes (Lycopene epsilon-cyclase, *ε-LCY*, and Lycopene beta-cyclase, *β-LCY*), key enzymes involved in carotenoid biosynthetic pathway [35]. High levels of carotenoid pigments in durum wheat are favored by breeders and consumers for wheat flour/end-product color and for human diet because of their nutritional properties. ε-LCY and β-LCY are respectively responsible for lutein and β-carotene (a provitamin A precursor) accumulation during endosperm development [35–37]. In contrast to *ε-LCY* genes (A and B genomes) that have been cloned and characterized [38], *β-LCY* genes are not well understood in the molecular structural genes. For the first time we optimized in durum wheat the TILLING procedure for an efficient SNP detection by DHPLC and we compared two low-cost screening methods: the most popular *Cel*I cleavage assay based on agarose gel detection and the reviewed DHPLC technique. The simplicity of the DHPLC technique will be demonstrated and it will make possible new applications of SNP-based markers for crop breeding programs.

## Methods

### Plant material

Seeds of the Italian durum wheat cultivar Marco Aurelio (high grain yield, excellent yellow index and protein content and gluten strength) were used for the EMS mutagenesis treatment. A set of nulli-tetrasomic lines (NT) of *Triticum aestivum* cv. Chinese Spring lacking group 3 and 6 chromosomes [39, 40] were used to assess the genome-specific primers.

### EMS mutagenesis and production of the wheat TILLING population

Marco Aurelio seeds were treated with EMS as reported by Uauy et al*.* (2009) [9]. To test the ‘kill curve’ , batches of 100 seeds each were treated with different EMS concentrations (0.00, 0.60, 0.70, 0.80, 0.90 and 1.00 %) at two exposure times (7 and 18 h) in the growth chamber. Based on the germination rate, the 18 h-treatment with the range of 0.70-0.85 % EMS was chosen to mutagenize ~32,000 seeds. The EMS-treated seeds (M_1_) were sown in the experimental field of University of Bari (Valenzano, Italy) in 2012. One spike from each M_1_ plant was collected and 10 M_2_ seeds were sown in the field to obtain the M_2_ generation. At the tillering stage, leaves for DNA extraction were collected from one random plant from each M_2_ progeny. M_3_ seeds from each M_2_ plant were harvested and stored at 4 °C under vacuum conditions.

### Genomic DNA extraction and pooling

Genomic DNA was isolated from leaves of individual M_2_ plants, using the Dellaporta et al*.* (1983) DNA extraction protocol. Approximately 100 mg of lyophilized leaves were placed in 2 mL single tubes containing 2 steel beads (4 mm) and tissues were crashed using the TissueLyser system (Qiagen) two times for 45 sec at 30 Hz. Genomic DNA was measured at spectrophotometer (Nano-Drop™ 1000, Thermo Scientific), diluted to a concentration of 90 ng/ml and checked on a 1 % agarose gel using λ DNA (Invitrogen) as a concentration reference. Then, M_2_ plant DNA was pooled four-fold in a 96-well format. 1,140 M_2_ plants from three M_2_ plant DNA subsets, derived from different EMS treatments (0.70, 0.75 and 0.80 %) (380 mutant plants per each), were used for the molecular screening for a total of three 96-well plates.

### Candidate genes and PCR amplification

The complete wheat coding sequences of the *ε-LCY* genes (A and B genomes) (GeneBank: EU649785, EU649786) were identified on NCBI (National Center for Biotechnology Information, [41]). For *β-LCY* gene, sequence information were partial available (GeneBank: AK334392, JN622196, FJ814767 and BT009216), and two of them were reported as *β-LCY* complete coding sequence (FJ814767 and JN622196). All these sequences were subjected to bioinformatic analysis via *Aegilops tauschii* genome sequence database [42] and CerealsDB site [43] to distinguish the A and B genome homoeologous copies, and via SoftBerry [44] to predict the gene structures. The final gene sequences were reported into the Additional file 1: Supplemental file S1.

Both target genes were analyzed by CODDLE software (Choose codons to Optimize the Detection of Deleterious Lesions, [45]) to identify the optimal target regions, and aligned in BLAST to highlight the differences between homoeologous sequences. All primer pairs were set using Primer3 [46] (Table 1). Based on observed genome differences, primer pairs were tested on DNA of Chinese Spring nulli-tetrasomic lines of chromosome groups 3 (N3A-T3B, N3B-T3D) and 6 (N6A-T6B, N6B-T6D).

**Table 1:** Primer pairs used to amplify the *ε-LCY* and *β-LCY* genes. The final two columns give the size of amplicons and the used high-throughput technology (*Cel*I/agarose gel or DHPLC method). The partial denaturation DHPLC temperatures are reported in brackets

Gene	Genome	Exon region	Primer	Sequence (5’-3’)	Amplicon size (bp)	Method
*β-LCY-6A*	A	1	F4f	AGCCCTACAACCCGGGA	861	*Cel*I/agarose gel
			PC63r	CCCATGAAGATCTTGAGA		
			F4f	AGCCCTACAACCCGGGA	464	DHPLC (65.8 °C)
			OP6r	GTGCGCGCCACCATGTACC		
*β-LCY-6B*	B	1	PC71f	ATCCCGGCCACCGTCGTCCTGGA	990	*Cel*I/agarose gel
			PC62r	CCATGAAGATCTTGAGATGC		
			PC68f	GTCTTCATCGACGACCACA	761	DHPLC (65.1 °C)
			OP6	GTGCGCGCCACCATGTACC		
*ε-Lcy-3A*	A	4–9	PC35f	TGCTGAGAAGGTAGACATTCTATTG	1,193	*Cel*I/agarose gel
			PC40r	CAAGCATTGATGGACTGGAC		
		4–5	PC35f^a^	TGCTGAGAAGGTAGACATTCTATTG	650	DHPLC (57.8 °C)
			PC130r^a^	CATTGCAGAAGCACACTGC		
		6–7–8	PC42f^a^	GGTTGATGTCTCGGTTGGAT	443	DHPLC (57.9 °C)
			PC40r^a^	CAAGCATTGATGGACTGGAC		
		8–9	PC43f	TGGACAATATTTGCCTGGAA	385	DHPLC (57.5 °C)
			PC37r	CTTGCGTACTCGCGAAAAA		
			PC35f	TGCTGAGAAGGTAGACATTCTATTG	1,527	Used for nested PCR
			PC37r	CTTGCGTACTCGCGAAAAA		
*ε-Lcy-3B*	B	4–9	PC42f	GGTTGATGTCTCGGTTGGAT	1,530	*Cel*I/agarose gel
			PC46r	GCATCCTTGCGTATTGTATTCTT		
		4	PC44f^a^	TTGCTGAGAAGGTACATTCGAT	336	DHPLC (58.4 °C)
			PC140r^a^	GGCACTTTGTGCAGGGTTGG		
		5–6–7	PC41f	GAGGACCACGTGTTTGTGTG	584	DHPLC (58.1 °C)
			PC143r	ACACCTGTGCAAGATAAACC		
		7–8–9	PC147f	TCCTTACCTAACACAGACCAGA	636	DHPLC (58.2 °C)
			PC48r	AAAGATACGCATCCTTGCGTATT		

^a^Primer combinations required nested PCR

Since we used two different pre-screening methods (*Cel*I/agarose gel and DHPLC), the primer combinations changed based on the technical requirements of each strategy. For the *ε-LCY-3A* and *ε-LCY-3B* genes, the CODDLE predicted target gene regions (exons 4*–*8) were longer than 1,600 bp. Thus, in *Cel*I/agarose system the amplicons covering exons 4*–*8 were obtained with the primer pairs PC35f-PC40r (1,193 bp) for *ε-LCY-3A* and PC42f-PC46r (1,530 bp) for *ε-LCY-3B* (Table 1). In DHPLC analysis, PCR primers for *ε-LCY* genes were designed dividing the target gene region into three parts: PC35f-PC130r, PC42f-PC40r and PC43f-PC37r amplicons for A genome and PC44f-PC140r, PC41f-PC143r and PC147f-PC48r amplicons for B genome.

On the other hand, the *β-LCY* gene regions analyzed through *Cel*I/agarose gel system corresponded to two amplicons of 861 bp in A genome (F4f-PC63r) and 990 bp in B genome (PC71f-PC63r). For DHPLC analysis, amplicon region sizes were limited to the first part of *β-LCY* exon 1 and precisely on 464 for A genome (F4f-OP6r) and 761 bp B genome (PC68f-OP6r). Even for *β-LCY* genes, genome-specific primers were assessed combining one specific genome primer with another one amplifying both homoeologous genome copies.

PCR reactions were carried out in a 20 μl volume, containing individual or pooled DNA, dH_2_O, 1X Phusion HF Buffer, 0.2 mM each dNTP, 0.3 μM each primer, 0.015 U Phusion High-Fidelity DNA Polymerase (Finnzymes). PCR reaction was conducted using a Bio-Rad thermocycler as follow: 98 °C for 30 sec for initial denaturation, followed by 30 cycles at 98 °C for 10 sec, an annealing temperature specific for each primer pair for 30 sec, 72 °C for 40 sec, one cycle at 72 °C for 10 min and 4 °C hold for storage. After PCR amplification, 3 μl of each amplified product were checked on 1 % agarose gel to verify the primer efficiency and the DNA concentration before the next TILLING step.

### *Cel*I nuclease digestion and mutation detection on agarose gel

Once the amplification and denaturing steps were done, 12*–*15 μl of PCR products (~600 ng) were digested with Surveyor *Cel*I enzyme (Transgenomic, Omaha, NE, USA). For each *Cel*I digestion, 1.2 μl of MgCl_2_ (0.15 mM), 1.2 μl of Enhancer Cofactor, 0.2 μl of Surveyor enhancer W and 0.2 μl of Surveyor nuclease were gently vortexed, added to PCR amplification and incubated at 42 °C for 60 min. Then nuclease digestion was ended with 2 μl of STOP solution and 3 μl of bromophenol blue loading dye for visualization on 1-2 % agarose gel.

The protocol for SNP detection method through *Cel*I/agarose gel has been applied according to Uauy et al*.* (2009) [9] with one modification: after each PCR reaction and agarose gel check, denaturing and re-annealing steps for heterduplexes formation were performed at 95 °C for 3 min.

### DHPLC mutation detection

The DHPLC analysis was performed using Transgenomic WAVE system (Transgenomic) equipped with a DNASep HT cartridge, solvent degasser unit, binary pump, autosampler (model 7250), oven (L-7310 T), accelerator and variable wavelength detector (UV/Vis/fluorescent). DNA fragments were separated using two buffers: buffer A (aqueous solution of 0.1 M TEAA, pH 7.0) and buffer B (aqueous solution of 0.1 M TEAA, pH 7 with 25 % (v/v) acetonitrile).

DHPLC system was tested before each plate runs by injecting the three molecular standards (Transgenomic): WAVE DNA Sizing Control (analyzed at 50 °C), WAVE High-Range Mutation Marker (70 °C) and WAVE Low-Range Mutation Marker (56 °C) according to the supplier’s recommendations. After each injection run, cleaning of the column was performed with buffer D (solution containing 75 % acetonitrile and 25 % water).

The preliminary *Cel*I digestion test in the Transgenomic WAVE system was carried out using two case–control wheat DNAs. Single homoduplex PCR products (PC133f-PC130r primers) were mixed in equal molarity in two-fold PCR pools and cleaved using 2 μl of *Cel*I. Each digestion was injected onto the WAVE system and either loaded on agarose gel to confirm the cleavage patterns. Amplicons were ran on DHPLC in Universal Linear gradient at 50 °C. The detected samples traces were compared in UV (260 nm) and in WAVE High Sensitivity System using the intercalating fluorescent WAVE Optimized HS Staining Solution. Putative mutations were identified by the presence of two chromatogram peaks whose size added up to the full length PCR product.

Molecular screening of the TILLING population was performed under the mutation detection Rapid DNA program. Amplicons were injected on the DHPLC and undergone partial denaturation method. Optimal temperature for SNP scanning was determined using DHPLC Navigator™ Software (Transgenomic) (Table 1). The column mobile phase consisted of different ratios of buffer A (ranging from 49-40 %) and buffer B (ranging from 51-60 %) in a mixture with varying acetonitrile concentrations based on the amplicon sequence and size.

Fourfold pools showing mutant profiles were opened and mixed in mutant DNA two-fold pools, heated and loaded again to identify the single plant carrying the mutation. DNAs of detected mutant lines were then sequenced by Sanger method.

## Results and Discussion

### Optimal mutagenesis conditions and development of the TILLING population

An optimal mutagen dosage is important to obtain individuals carrying a high mutation load with vigorous growth and fertility [13, 47, 48]. Therefore, to determine the concentration of mutagen treatment specific for the durum wheat cv. Marco Aurelio and to balance maximum mutation load with an acceptable plant survival rate, we carried out incubations for sets of 100 seeds with EMS concentrations ranging from 0.0 to 1.0 % at two different exposure times (7 and 18 h). As a large number of sulfur compounds, EMS exhibits high volatility and instability considering atmosphere oxidation and temperature [49]. According to our previous observation on the different EMS activities between batches, the following conditions were used: fresh EMS solution for each treatment step, controlled environmental temperature (25 °C) and a rotary shaker at a constant speed (112.5 rpm). After incubation and repeated washes, M_1_ treated seeds were sown, and germination rate was determined after six days (Table 2). As expected, the germination rates gradually decreased from 70 % to 10 % by increasing the EMS concentration from 0.60 to 1.00 %, respectively (both at 7 h and 18 h of incubation). A significantly different germination rate of 40 % and 28 % at 0.80 % EMS was observed at the two different time exposure.

**Table 2:** Germination rate (%) of Marco Aurelio seeds treated with different EMS concentrations and two exposure times (7 and 18 h). Results refer to 100 seed batches for each treatment in root-trainers in the growth chamber

Exposure time (h)	EMS concentration (%)					
	0.00	0.60	0.70	0.80	0.90	1.00
7 h	100	70	59	40	28	10
18 h	92	69	61	28	30	10

Seeds treated with 0.7 and 0.8 % EMS for 7 h showed values of 59 and 40 % of germination rate, respectively, in comparison with the untreated control (100 %), while a very low germination rate (10 %) was observed for the 1.00 % EMS treatment. After 18 h of incubation, the germination rate varied slightly between seed lots at 0.70-0.60 % EMS treatments and severely decreased for the 0.80-1.00 % EMS dosage (28 and 10 %, respectively). At 0.70 % EMS treatment for 7 and 18 h, similar value of germinability was observed. The comparison between the two experiments showed a slight decrement of seed vitality from 7 to 18 h treatment, as confirmed by the germination rates of the untreated seeds. Based on these results, we decided to use a range of EMS dosage (0.70-0.85 %) at 18 h treatment for a large-scale mutagenesis to obtain high mutation density and a survival rate of approximately 40 %.

Seeds were mutagenized with 0.70, 0.75, 0.80 and 0.85 % EMS for 18 h incubation and grown in field conditions. Out of 32,000 treated seeds, 6,745 M_1_ plants (21 %) produced filled spikes, while the rest died or produced sterile spikes (Table 3). The mortality rate was 77-80 % at 0.70-0.80 % EMS concentration, while a higher value of 88.7 % of mortality was observed with the 0.85 % EMS treatment. In total, at M_3_ generation we obtained 6,460 plants (0.70-0.85 % EMS population), including 2,000 lines treated at 0.70 % EMS dosage, 3,400 lines at 0.75 % EMS, 984 lines at 0.80 % EMS and 361 lines at 0.85 % EMS.

**Table 3:** Mortality rate (%) in M_1_ plants for each selected EMS concentration (%)

EMS (%)	Seed quantity (gr)	N° of seeds	N° of M_1_ plants	Mortality rate (%)
0.70	720	9,600	2,000	79.2
0.75	1,080	14,400	3,400	76.4
0.80	360	4,800	984	79.5
0.85	280	3,200	361	88.7
Total	2,440	32,000	6,745	78.9

### Candidate genes

Once the mutagenized durum population was available, a pilot assay was performed to determine the mutation frequency and the suitability of each TILLING population subsets. For this analysis, DNAs from a total of 1,140 plants (380 plants from the 0.70, 0.75 and 0.80 % EMS dosages) were pooled in groups of four DNAs and organized into three 96-well plates for convenient screening.

The genotypes were screened for the detection of mutations in two carotenoid genes: Lycopene epsilon-cyclase (*ε-LCY*) and Lycopene beta-cyclase (*β-LCY*). A region of ~1,300 bp for the *ε-LCY-3A* and *ε-LCY-3B* genes and one of ~800-900 bp for the *β-LCY-6A* and *β-LCY-6B* genes were selected for the pilot assay on the sub-set of M_2_ lines (Table 4). Amplicon size varied on the bases of the SNP detection method: in the *Cel*I assay based on agarose gel, we considered PCR products up 1,000 bp, while in the DHPLC method, we set the amplicon length with a maximum of 800 bp.

**Table 4:** Characterization of the *β-LCY* and *ε-LCY* genes: gene size (bp), number of exons and introns, the predicted regions by CODDLe program in terms of exon region and region length (bp) and the considered amplicon size for the TILLING strategy

Gene	Size (bp)	Number of exons	Number of introns	CODDLe prediction		TILLING approach
				Exon region	Length (bp)	Amplicon size (bp)
*β-LCY-6A*	1,703	1	0	1	400	880
*β-LCY-6B*	1,455	1	0	1	400	875
*ε-LCY-3A*	4,652	10	9	4–8	1,299	1,163
*ε-LCY-3B*	4,435	10	9	4–8	1,299	789

### Optimization of TILLING procedure through DHPLC

In order to test the performance of the DHPLC scanning procedure for SNPs in mixed PCR amplicons, a known polymorphism A- > G at 2,709 bp position of exon 5 of *ε-LCY-3A* was chosen for analysis in the case–control durum wheat genotypes cvs. Saadi and Casanova.

The DHPLC procedure involved the evaluation of two pre-screening methods of SNP detection: DHPLC combined to *Cel*I endonuclease digestion and DHPLC using melting temperature analysis. The DHPLC/*Cel*I optimization was carried out exploring data from UV and Fluorescence analysis. The *ε-LCY-3A* amplicon groups corresponding to PC133f-PC130r from cvs. Saadi and Casanova DNAs in equal molarity and in homoduplex status were mixed in two-fold PCR DNA, heated, cooled down to achieve heteroduplex formation and digested with 0.2 μl *Cel*I enzyme. The DHPLC/*Cel*I size criteria (universal linear gradient type application) were satisfied by the amplicons, which was ranging from 100 to 1,000 bp and a minimum distance of 50 bp between primers and SNP location.

**Fig. 1:**
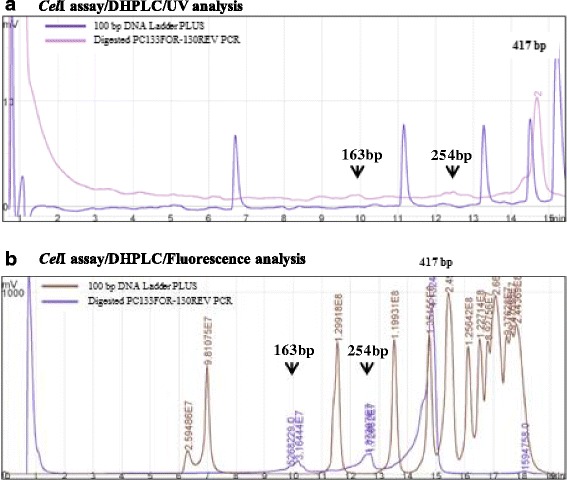
Comparisons of *Cel*I/DHPLC analysis using UV detector (**a**) and fluorescent detector (**b**). Chromatograms show the DHPLC run of PC133f-PC130r amplicons from mixed DNA. All runs are compared to 100 bp DNA Ladder PLUS. The black arrows indicate the putative expected heteroduplex molecules. The number above the main peak marked the region amplified length

The amplicon length in the DHPLC/*Cel*I chromatograms was in agreement with the PCR product size in both analysis, as confirmed by the presence of a single peak corresponding to the full-length fragment compared to the 100 bp DNA ladder PLUS. Regarding the heteroduplex cleavage profile (Fig. 1), the heteroduplex PC133f-PC130r amplicon showed the expected pattern of three peaks (two heteroduplex molecules corresponding to 163 bp and 254 bp peaks, and one homoduplex molecule of 417 bp) only in the fluorescence analyses. Even if the heteroduplex peaks were revealed and confirmed, the presence of SNP appeared as low traces (height peaks of ~40 mV compared to the homoduplex peak ~1,000 mV). The SNP detection signal could be covered by the PCR background noise (Fig. 1).

Generally, the issues to be taken into consideration for *Cel*I/DHPLC include the addition of *Cel*I reaction, the availability of a fluorescent detector (as here demonstrated to have major sensitivity in cleaved fragment resolution), the number of variable sites within the target region and the amplicon length (100–1,000 bp in size). The *Cel*I/DHPLC approach increases the economic costs and time of its application, since it requires a running time of 20 min/sample. The possibility of producing labmade *Cel*I endonuclease [50] could reduce costs but the sensitivity of the fluorescent detector still remains a limit, as confirmed by the noise level of the chromatograms due to interfered factors with the enzyme cleavage and their accumulation in the chromatographic column.

**Fig. 2:**
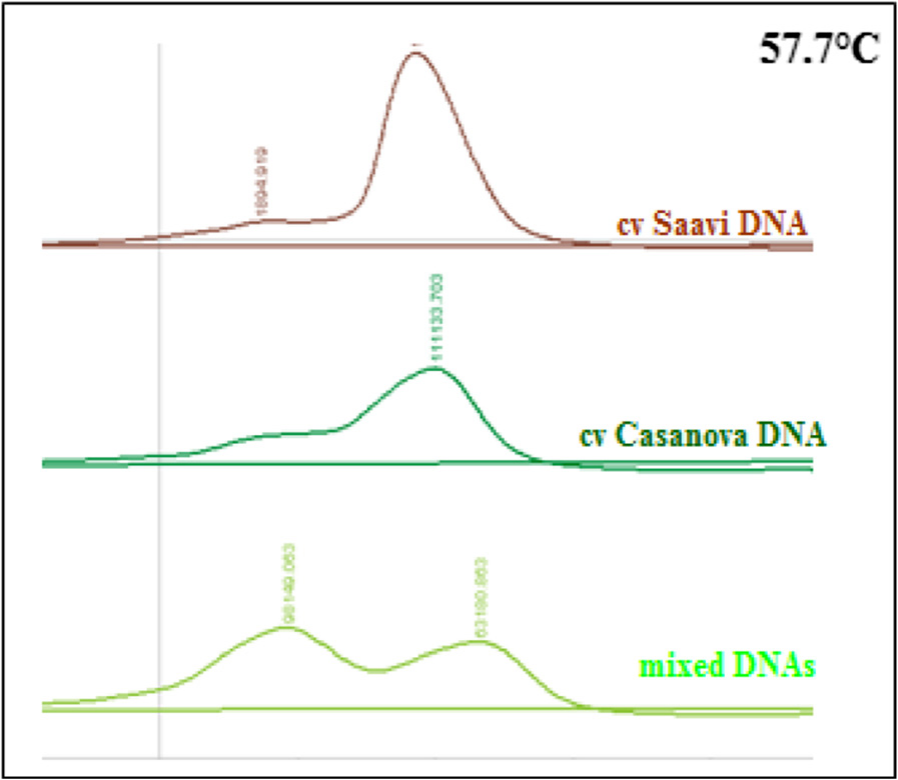
Outline of SNP detection by heteroduplex analysis of PC133f-PC130r amplicons of *ε-LCY-3A* gene. The chromatograms correspond to elution profile of homoduplex samples (cv. Saavi DNA in brown line and cv. Casanova DNA in dark green line) and the double peak of heteroduplex mixed DNA (green line). All runs were conducted at the same melting temperature in DNA Rapid program (57.7 °C)

Alternatively to the DHPLC/*Cel*I method, we considered a fast run time of 3 min through DHPLC based on the reduction of melting temperature between homo- and heteroduplex molecules. The criteria of DHPLC in melting temperature analysis consisted in high PCR product yields (~500 ng) and fragment size ≥ 200 and ≤ 800 bp. According to this, we focused on the melting profile of the PC133f-PC130r PCR product using the Navigator™ software (Transgenomic). It establishes whether any significant shifts in T_m_ could be predicted for the amplicon. The predicted melting temperatures (57.7 °C) was tested to determine the optimal run and heteroduplex visualization after denaturating/reannealing steps (Fig. 2).

The DHPLC-melting temperature analysis displayed to be more analytically sensitive and adaptable to a high-troughput format. The cost can be further reduced combining amplicons with different size but the same melting temperature profile in one run (under detection mutation parameters). Other considerations that should be considered to succeed with the DHPLC method are the fragment length (200–800 bp of size) and the presence of multiple thermal profiles that could require more than one temperature-gradient condition. All the candidate genes in the present work (*β-LCY* and *ε-LCY*) were optimized for heteroduplex detection in only one melting temperature.

**Fig. 3:**
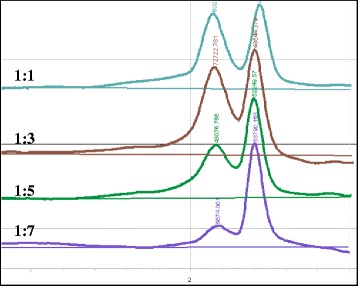
The pool depth setting for SNP detection. From the top line to the bottom, PC133f-PC130r PCR products from cvs. Saadi and Casanova are mixed together in proportions of 2, 4, 6 and 8 fold pools

The DHPLC protocol optimization also involved the adjustment of sample DNA concentration in terms of pool size. Exactly 12 μl of PCR products from cvs. Saadi and Casanova were mixed together in proportions of 1:1, 1:3, 1:5 and 1:7 (Fig. 3). As expected, a progressive reduction in SNP detection was observed with the increment of the pool level: the heteroduplex molecules were still visible up to the pool ratio of 1:5. The 1:3 pooling size was chosen for the M_2_ DNA arrangement.

### Induced mutation screening using the DHPLC and comparison with *Cel*I-agarose

The screening of the TILLING durum population included a pilot assay of DNAs arranged in four fold pools. According to Uauy et al*.* (2009) [9], the applied TILLING strategy was based on the initial screening of four-fold DNA pools to identify the mutant DNA and a second screening of two-fold DNA pools to identify the mutant individual for sequencing. The analysis was performed on the DHPLC technique using the melting temperatures defined in the optimization assay. Screening the *ε-LCY* and *β-LCY* genes, we identified a total of 46 mutations. The mutation density was calculated by dividing the total number of base pairs screened (this means the amplicon length multiplied by the total number of mutant lines) by the number of the detected mutations. The mutation density was estimated to be of 1/77 kb. Translating into single genes, we revealed 25 mutations for *β-LCY* genes (1,225 bp analyzed in total) and 38 mutations for *ε-LCY* (2,747 bp screened for A and B genomes) genes (Table 5).

**Table 5:** Mutation frequencies obtained through DHPLC analysis on 1,140 screened plants of the mutagenized durum wheat cv. Marco Aurelio

Gene	Primer		Exon region	Amplicon size (bp)	Mutation n°	Mutation frequency (1/kb)	
*β-LCY-6A*	F4f-OP6r		1	464	17		1/31
*β-LCY-6B*	PC68f-OP6r		1	761	8		1/108
*ε-LCY-3A*	PC35f-PC130r		4–5	650	12	1/62	
	PC42f-PC40r		6–7–8	443	2	1/252	
	PC43f-PC37r		8–9	384	3	1/146	
		mean					1/102
*ε-LCY-3B*	PC44f-PC140r		4	336	4	1/95	
	PC41f-PC143r		5–6–7	584	7	1/95	
	PC147f-PC48r		7–8–9	636	10	1/72	
		mean					1/84
Total/mean				4,258	46		1/77

We tried to simplify the SNP detection in TILLING population comparing results of two low cost procedures: DHPLC in melting parameters and *Cel*I-cleaved products on conventional agarose gel. Although the *Cel*I/agarose procedure is clearly a simplification of the TILLING technique, the efficiency of parameters were not so immediately obvious. We tested all the important parameters such as *Cel*I concentration, size of scanning windows and overall robustness of the method.

Using *Cel*I-agarose methods, we estimated a mutation frequency of 1/168 kb (31 mutations) for both genes. In particular, we yielded 11 and 20 mutations for *β-LCY* (1,861 bp screened in total) and *ε-LCY* (2,723 bp) genes, respectively (Table 6).

**Table 6:** Mutation frequencies obtained through *Cel*I/agarose method on 1,140 screened plants of the mutagenized durum wheat cv. Marco Aurelio

Gene	Primer	Exon region	Amplicon size (bp)	Mutation n°	Mutation frequency (1/kb)^a^
*β-LCY-6A*	F4f-PC63r	1	871	5	1/176
*β-LCY-6B*	PC71f-PC62r	1	990	6	1/167
*ε-LCY-3A*	PC35f-PC40r	4–8	1,193	9	1/138
*ε-LCY-3B*	PC44f-PC46r	4–8	1,530	11	1/148
Total/mean			4,584	31	1/168

^a^The fragment size of each gene was reduced to 100 bp because of PCR artefacts

Overall, the highest number of identified mutations was observed in the conserved region of 464 bp size of *β-LCY-6A* gene (17 mutations) and in the ~600 bp regions of *ε-LCY-3A* amplicon 1A and *ε-LCY-3B* amplicon 3B (12 and 10 mutations, respectively). Each method identified 26 heterozygous loci detected by the other one. All mutations were confirmed by direct sequencing. The mutant genotypes were in heterozygous state, excepting for 12 homozygous mutations in in *ε-LCY-3A* and *ε-LCY-3B* genes. As expected because of the EMS alkylation, all of induced mutations were G to A or C to T transitions.

The 31 mutations (corresponding to 1/168 kb) detected on agarose gel could give a wrong consideration of the mutation density rate of the durum TILLING population. Unlike that of the DHPLC genotyper, the sensitivity of SNP detection on agarose gels is low although it can be sufficient to test only the presence/absence of SNPs in small DNA populations.

## Conclusions

The results obtained by the use of DHPLC in melting temperature conditions demonstrated this method is an excellent alternative approach for mutation screening in wheat TILLING populations. Although DHPLC requires an initial investment in terms of instrument, consumable and eventually *Cel*I endonuclease kit, it proved to be outsourced from a direct sequencing service, less expensive and more sensitive than cleavage assay based on agarose gel. Finally, this is the first report of TILLING study in durum wheat, providing good evidence of DHPLC applicability and potentiality in SNP detection in target genes. The availability of these tool, together to the continue increase number of SNP markers by genotyping array services, could expand the use of SNP markers in durum wheat breeding programs.

## Additional file

Additional file 1:
**Supplemental file S1.** β-LCY gene sequences. (DOCX 12 kb)

## Abbreviations

CODDLE: choose codons to optimize the detection of deleterious lesions
DGGE: denaturing gradient gel electrophoresis
DHPLC: denaturing high performance liquid chromatography
EMS: ethyl methane sulphonate
EST: expressed sequence tags
HRM: High Resolution DNA Meltin
MS: Mass Spectrometry
NGS: next-generation sequencing
PCR: polymerase chain reaction
SSCP: single-strand conformational polymorphism
SNP: single nucleotide polymorphism
TILLING: targeted induced local lesions in genomes
TbyS: TILLING by sequencing
ε-LCY: lycopene epsilon-cyclase
β-LCY: lycopene beta-cyclase
