# Supplementary material for: DHPLC technology for high-throughput detection of mutations in a durum wheat TILLING population
Source: BMC Genet. 2016 Feb 17;17:43. doi: 10.1186/s12863-016-0350-0 (PMC4756519; doi:10.1186/s12863-016-0350-0)
Supplement: Additional file 1: — Supplemental file S1. β-LCY gene sequences. (DOCX 12 kb) [file 12863_2016_350_MOESM1_ESM.docx]

**Supplemental File S1:** *β-LCY* ***gene sequences***

The *β-LCY* gene sequences (A and B genomes) are provided as classical FASTA files.

**>** β-LCY-6A

AGCCCTACAACCCGGGATACCAGGTCGCCTACGGCATCCTCGCCGAGGTCGACGAGCACCCCTTCGACATCGACAAGATGCTCTTCATGGACTGGCGCGACTCCCACCTCCCCGAGGGCTCCGCGATCAAGGAGCGGAACAGCCGCGTGCCCACCTTCCTCTACGCCATGCCCTTCTCGCCCACCAGGATCTTCCTCGAGGAGACGTCGCTGGTCGCGCGCCCGGGGCTCTCCATGGACGACATCCAGGAGCGCATGGCCGCGCGGCTGAGGCACCTGGGAATCCGCATCCGGAGCGTCGAGGAGGACGAGCGCTGCGTGATCCCCATGGGCGGGCCGCTGCCCGTGCTGCCGCAGAGGGTGGTGGGCATCGGCGGCACGGCCGGGATGGTGCATCCGTCCACAGGGTACATGGTGGCGCGCACATTGGCGACCGCGCCCATCGTGGCAGACTCCATCGTGCGGTTTCTAGACACCGGCAACGGCGGCATCGCCGGGGACGCGCTCGCCGCCGAGGTGTGGAAGGAGCTGTGGCCGACGGACAGGCGGCGGCAGAGGGAATTCTTCTGCTTCGGCATGGACGTCCTGCTCAAGCTGGACCTCCAAGGTACACGACGGTTCTTCAACGCATTCTTCGACCTCGAGCCGCACTACTGGCACGGCTTCCTCTCGTCGAGGCTGTTCCTGCCTGAGCTCTTGATGTTTGGGCTCTCGCTGTTCGCGCACGCTTCCAACACGTCCAAGCTGGAGATCATGGCCAAGGGCACCGTGCCTCTTGCCAAGATGGTCGGCAACTTGATACAGGACAAGGATAGGTGATGACTTAGAGGGTATGTATGTACCTGCATTTCTCATCTCAAGATCTTCATGGG

**>** β-LCY-6B

ATCCCGGCCACCGTCGTCCTGGACGCCACCGGCTTCTCCCGCTGCCTCGTGCAGTACGACAAGCCCTACAACCCGGGCTACCAGGTCGCCTACGGCATCCTCGCCGAGGTCGACGAGCACCCCTTCGACATCGACAAGATGCTCTTCATGGACTGGCGCGACTCCCACCTCCCCGAGGGGTCCGCGATCAAGGACCGGAACAGCCGCGTGCCCACCTTCCTCTACGCCATGCCCTTCTCGCCCACCAGGATCTTCCTCGAGGAGACGTCGCTGGTCGCGCGCCCGGGGCTCTCCATGGACGACATCCAGGAGCGCATGGCCGCGCGGCTGAGGCACCTGGGAATTCGCATCCGGAGCGTCGAGGAGGACGAGCGCTGCGTGATCCCCATGGGCGGGCCGCTGCCCGTGCTGCCGCAGAGGGTGGTGGGCATCGGCGGCACGGCCGGGATGGTGCATCCGTCCACAGGGTACATGGTGGCGCGCACATTGGCGACCGCGCCCATCGTGGCAGACTCCATCGTGCGGTTTCTAGACACCGGCAACGGCGGCATCGCCGGGGACGCGCTCGCCGCCGAGGTGTGGAAGGAGCTGTGGCCGACGGACAGGCGGCGGCAGAGGGAATTCTTCTGCTTCGGCATGGACGTCCTGCTCAAGCTGGACCTCCAAGGTACACGACGSTTCTTCAACGCATTCTTCGACCTCGAGCCGCACTACTGGCACGGCTTCCTCTCGTCGAGGCTGYTCCTGCCTGAGCTCYTGATGTTYGGGCTCTCGMTGTTCGYGCACGCTTCCAACACGTCCAAGCTGGAGATCATGGCCAAGGGCACCGTGCCTCTTGCCAAGATGGTCGGCAACTTGATACAGGACAAGGATAGGTGATGACTTAGAGGGTATGTATGTACCTGCATTTCGCATCTCAAGATCTTCATGG
